# Supplementary figures and images for: Determining the interaction status and evolutionary fate of duplicated homomeric proteins
Source: PLoS Comput Biol. 2020 Aug 27;16(8):e1008145. doi: 10.1371/journal.pcbi.1008145 (PMC7480870; doi:10.1371/journal.pcbi.1008145)

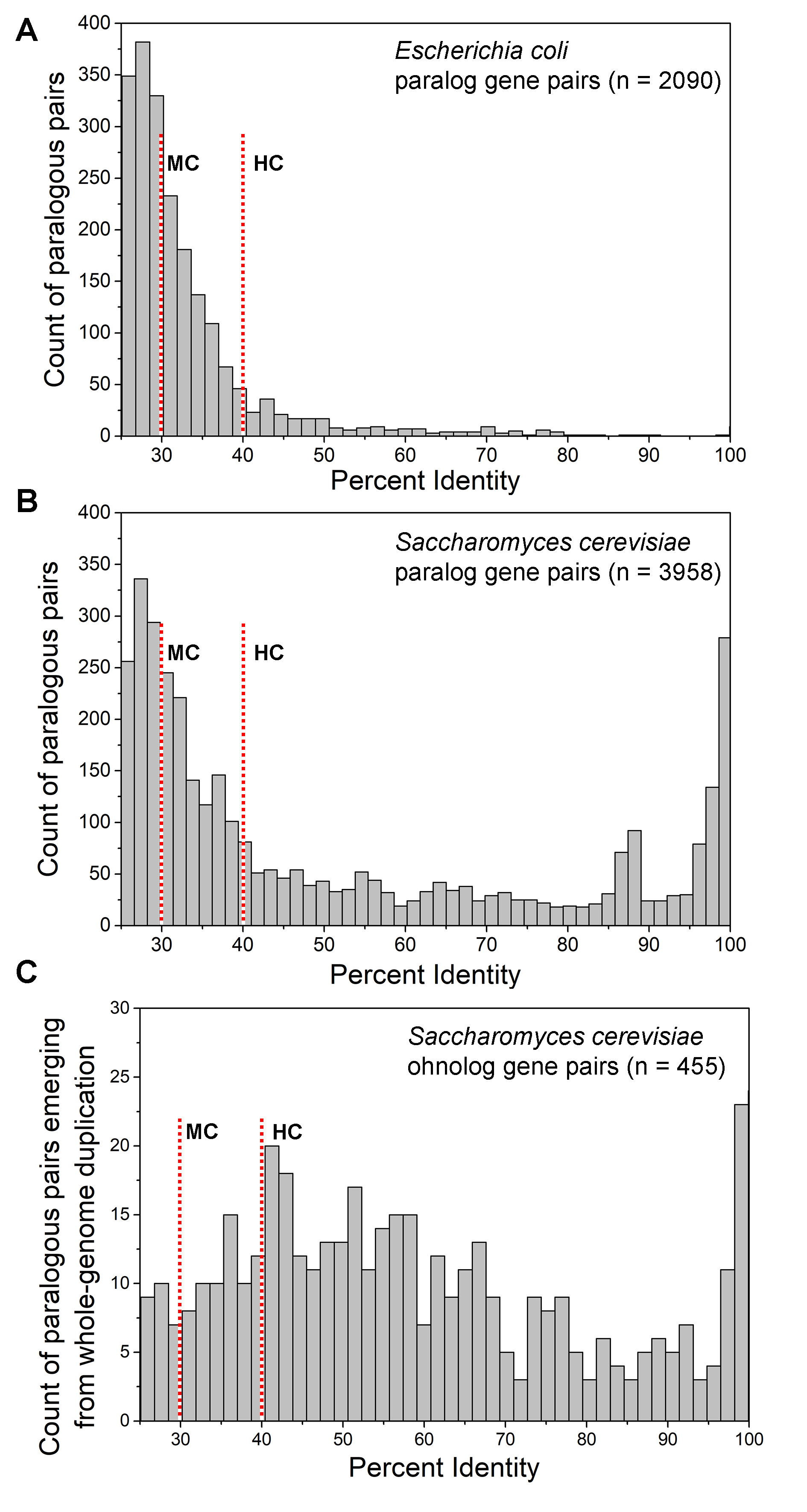

Supplement: S1 Fig — (A) E. coli paralogs (n = 2090 pairs). (B) S. cerevisiae all paralogs (n = 3958 pairs). (C) S. cerevisiae ohnologs (the subset of paralogs that arose from the whole genome duplication; n = 455 pairs). Note that these plots include all paralogs, not only the ones for which molecular interaction data are available. The dotted red lines represent the identity thresholds used for defining MC (≥30% identity) and HC (≥40% identity). (TIF) [file pcbi.1008145.s001.tif]
