# Supplementary material for: Determining the interaction status and evolutionary fate of duplicated homomeric proteins
Source: PLoS Comput Biol. 2020 Aug 27;16(8):e1008145. doi: 10.1371/journal.pcbi.1008145 (PMC7480870; doi:10.1371/journal.pcbi.1008145)
Supplement: Attachment — Submitted filename: Revision letter.docx [file pcbi.1008145.s008.docx]

| / | **Weizmann Institute of Science**  Rehovot 76100, Israel | **Dan S. Tawfik, Ph.D.**  The Nella and Leon Benoziyo Professor  Department of Biomolecular Sciences  Phone: +972 8 934-3637  Fax: +972 8 934-4118  Email: dan.tawfik@weizmann.ac.il |
| --- | --- | --- |

21 June, 2020

The Editors

*PLoS Computational Biology*

***Re: Revision of PCOMPBIOL-D-20-00771***

Dear Drs. Elofsson, Shakhnovich,

Thank you very much for considering our submission, and for the editor’s and the two reviewers’ supportive and constructive comments. These have all been address as detailed below. The additions and changes to the manuscript are highlighted in red.

We trust that you will find the revised version suitable for publication.

Thanks again, best regards,

Dan, Saurav

**PCOMPBIOL-D-20-00771 - Point-by-Point response**

**Original comments are in blue and responses in black**

------------------------------------------------------------------------

**Editor’s comment**

This paper provides valuable insights and we will be interested to consider it further after reviewers’ suggestions are addressed in the revised version. One more comment: the homo- vs hetero- oligomerization paths for dimer formation were carefully considered in an earlier work Lukatsky et al JMB (2007) v.365 pp.1596-1606. This work should be cited and discussed in the present manuscript.

**Response**

Thank you for this suggestion. In the revised manuscript, the above-mentioned paper is cited as ref. [6] and discussed in Page 3, lines 9-11, and in Page 10, lines 16-18.

------------------------------------------------------------------------

**Comments of Reviewer-1**

Mallik and Tawfik present a valuable computational study on the fate of duplicated oligomers. Duplications of oligomers initially lead to the formation of hetero-oligomers, and this initial situation can subsequently resolve into a variety of different scenarios, including obligate assembly into heteromers, assembly into only homomers, and losses of interfaces. Previous studies have variously estimated the frequency of these different fates, using high throughput interactomics data. This is associated with a number of biases and difficulties that Mallik and Tawfik address in this paper. One difficulty that remains (which all prior work also suffers from), is that using databases from a small number of model organisms makes it impossible to make explicit, polarized evolutionary inferences about changes in biochemical phenotypes. Nevertheless, this manuscript substantially improves on prior attempts to answer this question using similar methods. I consider this a paper valuable contribution to this question that moves the field forward.

The authors’ methodology is solid and the conclusions well supported as far as I can tell. My only minor comment is that perhaps Finnigan et al 2012 could be cited when the authors mention in the discussion that subfunctionalization of interfaces can aid the preservation of genes after duplication, as this idea has been articulated (and demonstrated experimentally) before.

**Response**

Thank you for this suggestion. The above mentioned paper is cited in the revised version as ref. [32] (see Page-10, Line-26).

------------------------------------------------------------------------

**Comments of Reviewer-2**

**Comment - 1.** An interesting study on the evolution of protein complex formation which will add to our understanding of the importance of gene duplication to our current understanding of systems biology. I would be interested in the answers to the following questions:

The statement about the reliability of Y2H as a methodology is now 18 years out of date, according to the reference used and would certainly be challenged by groups such as Vidal or Uetz. The authors either need to find more up-to-date evidence to support that statement or qualify it to make a more current assessment of this technology.

**Response**

We could not find an up to date assessment of the noise in Y2H. However, assessing the false-positive or false-negative rates in Y2H or pulldown experiments is not our main focus. Hence in the revised version, the above-mentioned text has been revised to only mention that high-throughput PPI data tend to be noisy and data filtering would influence the results (see Page-4, Line-28).

**Comment - 2.** What is the authors’ definition of 'high-throughput' PPI data? Whilst there is certainly plenty of these in both IntAct and BioGriD, both of these resources also contain significant amounts of low-throughput data, by most definitions - was this not used and, if not, then why not. The authors otherwise are largely limiting themselves to Y2H data for the identification of homomeric interactions, which seems strange if they really believe it is of poor quality.

**Response**

We thank the reviewer for pointing out that a clarification is needed. By ‘high-throughput PPI data’ we mean PPI data collected from databases that includes both high- and low-throughput data, with the former dominating. Note, however, that in effect, due to the filtering we applied, the high-throughput dominated (see comment 4 below).

To clarify this issue, a sentence was added to the Results (see Page-5, Line-3) and one to the Methods section (see Page-12, Lines 7-9).

**Comment - 3.** For all the resources used, a list of release numbers/download dates would have been useful when I tried to understand how the data had been used.

**Response**

Thank you for pointing this out. The release numbers and download dates from different molecular interaction databases are now listed in the revised legends of Data S1 and Data S2.

**Comment - 4.** The dataset filtering uses the bait-prey relationship. This is fine if the authors have limited themselves to Y2H and pulldown expts, but if they used other valuable data sources such as gel filtration and comigration in gels, how did they deal with data types which do not have this relationship.

**Response**

Thank you for pointing this issue out – this point had indeed escaped our attention. The reviewer is right that the bait-and-prey filtering criterion excluded interactions detected by any method where this is irrelevant (gel filtration, etc.). We added a sentence in the revised text to clarify this issue (Page-12, Lines 14-15).

To see if the assignment of homo/hetero fates in the PPI set was substantially influenced by this criterion, we extracted the subset of yeast PPI data that was exclusively detected by methods other than Y2H and pulldown. For this set, applying the *stringent criterion*, we assigned the homo/hetero fates of paralogous pairs and detected only 14 new pairs, in comparison to 1152 pairs enlisted in **Data S3**. Further, even in this small set, the trend of dominance of heteromers was reproduced. These 14 new pairs comprise 5 obligatory hetero, 7 mixed, 2 hetero others, and no obligatory homo. At this stage, we opted to avoid disturbing the entire datasets and **Figure 2**, as this can result in errors. These 14 new pairs are therefore listed as a new Supplementary data file: **Data S6**. This issue is discussed in the Methods section of the revised manuscript (see Page-13, Lines 12-20).

**Comment - 5.** The authors had to skip the lack of colocalisation filter step for Ecoli - did they look at using Gene Ontology data for this. Or the colocalisation data in the IMEx databases. There may not be enough information in either case, but it would be interesting to know if the authors had considered this.

**Response**

For *E. coli* the localization data is relatively scarce, and the overall number of pairs is relatively small compared to yeast. Hence, since for yeast applying the localization criterion did not make much difference with respect to the distribution of homo/hetero fates, we did not insist it for *E. coli*.

**Comment - 6**. The authors describe 7 different databases as 'curated' - this is fact not true. iREFIndex and STRING compile manually curated data from BioGRID and the IMEx data held in IntAct but do not add any manually curated data themselves whilst MENTHA is a browser which visualizes IMEx and BioGRID data. HiNT do at least add PDB data but I am not clear what was additionally gained from iREFIndex, STRING or Mentha. If STRING predictions/text-mining data was used, this should be made clear by the authors.

**Response**

Perhaps we have not been sufficiently clear. The word ‘curated’ in our manuscript is exclusively applied to macromolecular complexes, not to the PPI data. Rather, we used ‘curated’ to describe the data that came from the Protein Data Bank, or from Complex portal database, that was manually curated from various experimental sources. We have now checked the manuscript throughout to ensure the above.

Regarding the overlap. The different databases collect their raw data from published literature, and there is indeed considerable overlap between them, but no complete overlap. It is true, however, that the demand that the interaction must be reported in at least two databases does not necessarily mean two independent observations, but as a minimum it ensures that there is no annotation mistake. We clarified this issue in the revised manuscript (see Page-12, Lines 18-20).

Only experimentally verified interactions were included in our analysis. Thus, we did not use STRING-predicted text-mining based PPI data. We added a sentence in the revised text to clarify this (see Page-12, Line 9).

**Comment - 7**. Looking in the PSICQUIC viewer, the Rajagopala dataset is in IntAct and therefore also in Mentha and irefIndex and presumably also STRING. Why do the authors treat that set separately -it would surely be better to run it through whatever data filters were used for the interaction database sets.

**Response**

Yes, there is a large overlap between Rajagopala dataset and IntAct/BioGrid. Still, we found that Rajagopala dataset has 766 unique binary PPI data that are not included in IntAct and BioGrid. Therefore we believe this dataset should be considered separately.

**Comment - 8**. There does not seem to have a list of complexes used for Ecoli, only the proteins.

**Response**

In **Data S2** we do have complexes, not proteins. Perhaps the arrangement in sheets is confusing, yet Sheet# ‘PDB complexes’ includes a list of both homomeric and heteromeric complexes that comprise paralogous proteins. Homomeric complexes cover the first 88 lines of the sheet, which is probably why the reviewer thought it is a list of proteins.

**Comment - 9**. I was wondering why the authors converted all the Yeast information to yeast gene identifiers rather than leave them as UniProt protein IDs, which would make them more easily comparable with the E.coli data for anyone trying to work with the data. As UniProt seems to be the identifier in common used by most of the the resources listed, including Pfam, it seems an unnecessary extra step and could lead to data loss.

**Response**

We used the yeast gene identifiers because it is consistent with the SGD database which is the largest resource of yeast genomic/proteomic data and also of deletion strains. We have now revised Data S1 and S3 to also include the UniProt identifiers.
